# Differentiating Multisystem Inflammatory Syndrome From Neonatal Sepsis: A Case Report

**DOI:** 10.7759/cureus.69685

**Published:** 2024-09-18

**Authors:** Aditi Rawat, Sagar Karotkar, Mahaveer S Lakra, Ravi Reddy, Amar Taksande

**Affiliations:** 1 Neonatology, Jawaharlal Nehru Medical College, Datta Meghe Institute of Higher Education and Research, Wardha, IND; 2 Pediatrics, Jawaharlal Nehru Medical College, Datta Meghe Institute of Higher Education and Research, Wardha, IND

**Keywords:** mis-c, mis-n, multisystem inflammatory syndrome, neonatal sepsis, sars-cov-2

## Abstract

Severe acute respiratory syndrome coronavirus 2 (SARS-CoV-2) exhibits a spectrum of clinical manifestations, spanning from asymptomatic carriage to fatal outcomes. Among young infants, the incidence of severe disease is notably high. The pathogenesis of multisystem inflammatory syndrome (MIS) in neonates associated with SARS-CoV-2 remains elusive, although post-infective immune dysregulation is posited as a significant contributor. Recent cohorts have highlighted the transplacental transfer of immunoglobulins, potentially exacerbating immune dysregulation due to the co-transfer of inflammatory cytokines. Antenatal transmission of viral particles in neonates is rare, with suspicion of nosocomial infection in most cases. This abstract summarizes a case study of a neonate with MIS, presenting with cardiovascular, respiratory, and gastrointestinal involvement, along with fever and elevated biomarkers. Notable observations from similar cases include a predominance of cardiovascular and respiratory symptoms, albeit with variability in echo findings.

## Introduction

The severe acute respiratory syndrome coronavirus 2 (SARS-CoV-2) epidemic, which is more common in countries with dense populations like India, has shocked the medical world. Multisystem inflammatory syndrome (MIS) in children is a well-known illness in pediatrics, despite the fact that it is rarely seen in infants. At the beginning of the outbreak, there was little evidence to establish the transplacental transmission of SARS-CoV-2 from mother to fetus [1]. This method of transmission cannot be ruled out at this time because neonates delivered to mothers with a history of COVID-19 disease or exposure present with a hyper-inflammatory syndrome-like presentation that is identical to that of adults. MIS in neonates can mimic common disorders such as birth asphyxia and newborn sepsis; hence, a strong index of suspicion is needed to diagnose it [2]. Immunoglobulins are generally thought to be protective against SARS-CoV-2 infection, but the transplacental transfer of immunoglobulins, along with the in-utero transfer of other inflammatory cytokines, may mimic a process similar to MIS in children, potentially causing immune activation and manifesting as MIS in neonates [3]. Seroconversion response to SARS-CoV-2 infection has been seen within two to three weeks of symptomatic infection, with both immunoglobulin M (IgM) and immunoglobulin G (IgG) levels detected in plasma. Here, we describe a newborn case of late-onset sepsis with multiorgan involvement that did not improve with medical intervention. There was a discernible improvement after being classified as MIS in neonates and receiving treatment. As demonstrated in this case, it is critical to keep an eye out for suspicious MIS in neonates, as doing so could potentially save lives.

## Case presentation

A 30-year-old primigravida with well-controlled gestational diabetes gave birth to a 3.5-kilogram male baby boy via normal vaginal delivery. After birth, the baby cried, and no resuscitation was necessary. The father was vaccinated against COVID, but the mother was not. Pregnancy-related COVID-19 symptoms were absent in both parents. There were no maternal risk factors for sepsis in the newborn. After birth, the infant was moved to the mother's side. From the first hour of life, breastfeeding was initiated. Throughout the hospital stay, the baby remained euglycemic due to blood glucose monitoring. After being born, the infant was referred to our hospital because he had become lethargic and was having trouble receiving feeds.

The infant was sluggish, tachypneic (65 breaths per minute), tachycardic (heart rate 168 beats per minute), and febrile (temperature above 38°C) at the time of admission. The baby had hypoglycemic seizures, and random blood sugar was 30 mg/dL. Upon admission, the infant also presented with respiratory difficulty and grunting, for which continuous positive airway pressure (CPAP) was utilized. Suspecting late-onset sepsis, intravenous antibiotics were started, and parenteral glucose infusion was used as directed to treat symptomatic hypoglycemia. Despite a normal initial septic screen, the patient's admission C-reactive protein (CRP) level was elevated (29.72 mg/dL).

Cerebrospinal fluid examination was done to rule out meningitis, which was normal. The baby had a low-grade fever of 38-39°C throughout the stay, so antibiotics were upgraded. At 84 hours of life, the baby had one episode of gastric bleed, with a platelet count of 127,000 cells/cumm and a normal coagulation profile. By 96 hours of life, the baby developed persistent tachycardia associated with weak peripheral pulses and prolonged capillary refill time, which was managed by fluid bolus and inotropic support, suspecting septic shock.

**Figure 1:**
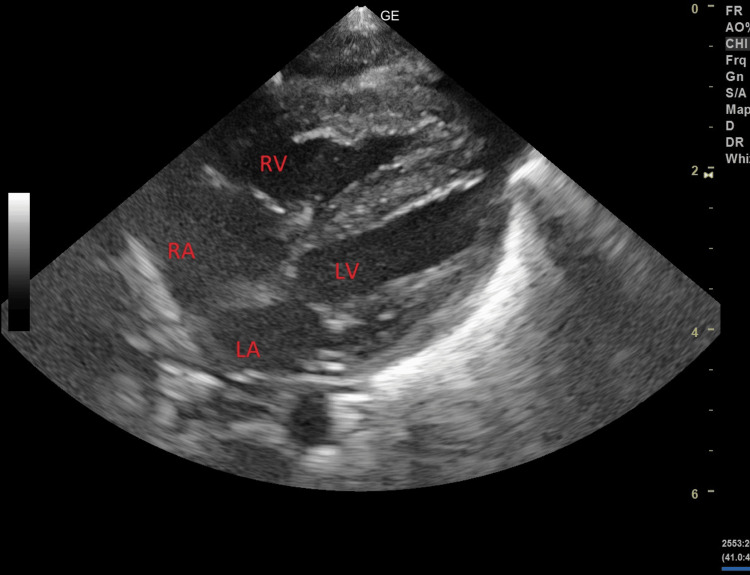
2D echocardiography, subcostal four-chamber view, showing biventricular and septal hypertrophy LA: Left atrium; LV: Left ventricle; RA: Right atrium; RV: Right ventricle

There was a subsequent improvement in pulses and capillary refill, with adequate urine output, but the tachycardia persisted. Inotropic support was gradually tapered and omitted, but the tachycardia persisted. The baby continued to have multiple fever episodes despite broad-spectrum antibiotic coverage. On day 5, the baby’s respiratory distress increased, for which he was mechanically ventilated. For persistent tachycardia, an electrocardiogram (ECG) and 2D echocardiography were done. The ECG was suggestive of sinus tachycardia with ST changes. The 2D echocardiography showed mild biventricular and septal hypertrophy (Figure 1).

Cardiac enzymes were raised as well, suggestive of myocarditis. For this unusual constellation of symptoms in a term baby - such as gastric bleed, myocarditis, hemodynamic instability, and respiratory distress requiring mechanical ventilation - a possibility of MIS in neonates was considered. To support the diagnosis of MIS in neonates, repeat CRP (106 mg/dL), D-dimer (30,174 ng/mL), pro-brain natriuretic peptide (4,040 pg/mL), aspartate aminotransferase (108 U/L), alanine transaminase (418 U/L), and serum ferritin (509 ng/mL) were done, all of which were significantly elevated. Renal functions were normal. Intravenous immunoglobulin (IVIg) was given at a dose of 1 gm/kg, in two doses 24 hours apart, following which the tachycardia settled and a drastic improvement in clinical condition was seen. Thereafter, the baby was gradually weaned off respiratory support (Table 1).

**Table 1:** Laboratory values day wise

Laboratory investigations	Day 1	Day 5	Day 10	Biological reference range
Hemoglobin	14.8	11.4	11.0	13-15 g/dL
Total leucocyte count	8,000	9,900	8,500	4,000-11,000 /cumm
Platelet	174,000	127,000	180,000	150,000-450,000 /cumm
C-reactive protein	29	106	28	0-1 mg/dL
Aspartate aminotransferase	-	108	-	8-33 U/L
Alanine transaminase	-	418	-	4-36 U/L
D-dimer	-	30,174	4,704	<500 ng/mL
Pro-brain natriuretic peptide	-	4,040	-	<100 picograms per milliliter (pg/mL)

Covid serology of the baby was sent, which came out to be positive with anti-SARS-CoV-2 IgG 19 AU/mL. Maternal and paternal COVID-19 serology was sent as well, which showed elevated IgG levels. Nasopharyngeal swab samples from the baby and both parents were sent for COVID-19 real-time reverse transcription polymerase chain reaction (RT-PCR), which came out to be negative. Antibiotics were stopped by the seventh day of life, as the blood culture was sterile. The baby was on full feeds by the ninth day of life. Sequential D-dimer and CRP showed a progressive fall to 4,704 ng/mL and 28 mg/dL, respectively. The baby was discharged by the 14th day of life. A final diagnosis of late-onset MIS in neonates presenting as myocarditis was made using the WHO diagnostic criteria, along with supportive evidence as mentioned above [3].

## Discussion

SARS-CoV-2 has a wide range of clinical presentations, ranging from asymptomatic to fatal. The incidence of severe disease in children <1 year of age is 10.6% [4]. The exact mechanism of MIS in neonates is still unclear. There is supposed to be an important role of post-infective immune dysregulation in neonates [5,6]. From the recent cohort of cases with MIS in neonates, we now know that the transplacental transfer of IgG antibodies does occur. These immunoglobulins, which are thought to be protective, may cause dysregulated immune activation due to the co-transfer of other inflammatory cytokines [6].

In neonates, there have been very few cases that show the transfer of virus or viral particles antenatally. Case series conducted by Liu et al. [7], Chen et al. [8], and Fan et al. [9] showed that the virus was not detected in the neonate’s nasopharyngeal swab samples at birth, in the placenta, umbilical cord, amniotic fluid, or in the maternal vaginal swab. Only two cases, to our knowledge, have demonstrated SARS-CoV-2-positive pharyngeal swabs within 48 hours of life [10,11]; the rest of the known cases in the literature were positive after 48 hours, which gives a high suspicion of nosocomial infection rather than transplacental. Our case had negative IgM and RT-PCR for SARS-CoV-2 done on the fifth day of life, although IgG was positive. In diagnosed cases of MIS in neonates, IgG positivity is found to be 73.3% according to Lakhkar et al. [12], 85% according to Pawar et al. [13], and 100% according to More et al. [2].

Our case presented with involvement of the cardiovascular system, respiratory system, gastric bleed, and fever with elevated biomarkers. A similar observation was made by More et al. [2], in which late MIS in neonates had 40% respiratory, 30% cardiac, and 70% febrile symptomatology. Case series by Pawar et al. [13] reported 90% cardiac involvement, 55% respiratory involvement requiring positive pressure ventilation, 10% gastric bleed, and 10% fever. A systematic review by Trevisanuto et al. [14] showed the clinical profile of MIS in neonates as follows: fever (50%), gastrointestinal symptoms (26%), hypoxia (20%), and cough (20%).

The commonly encountered echo findings, such as dilated coronaries, pericardial effusion, and left ventricular dysfunction, were not seen in this case [6,13]. Only biventricular hypertrophy was noted. Arrhythmias were a predominant feature, and a similar observation was made by Pawar et al. [13]. Recent evidence suggests that recovery from MIS in children is similar after primary treatment with IVIg alone, IVIg plus glucocorticoids, or glucocorticoids alone [15]. Treatment with IVIg alone showed dramatic improvement in our case.

## Conclusions

This case details a term neonate presenting with an unusual constellation of symptoms, including lethargy, hypoglycemic seizures, tachycardia, respiratory distress, and gastrointestinal bleeding. Initial concerns for late-onset sepsis were addressed with broad-spectrum antibiotics, but clinical deterioration and persistent symptoms led to further investigation. A diagnostic workup revealed myocarditis, elevated inflammatory markers, and positive COVID-19 serology, supporting a diagnosis of MIS in children. Treatment with IVIg resulted in significant clinical improvement. The baby eventually recovered and was discharged on day 14, and the final diagnosis of late-onset MIS in children presenting as myocarditis was confirmed. This case underscores the importance of considering MIS in neonates with severe, unexplained symptoms, especially in the context of recent or remote COVID-19 exposure. The numbers of symptomatic cases have decreased after the third wave. Hence, the clinician should have a keen eye on the clinical features of MIS in neonates for early recognition of such cases, even in the absence of a maternal history of COVID-19. MIS in neonates can have a wide spectrum of presentation, and after ruling out common differentials, workup of COVID-19 should be mandated in a rapidly deteriorating neonate. Timely and accurate diagnosis of MIS in neonates can be life-saving in critical cases.
